# Co-registration Analysis of Fluorodopa and Fluorodeoxyglucose Positron Emission Tomography for Differentiating Multiple System Atrophy Parkinsonism Type From Parkinson's Disease

**DOI:** 10.3389/fnagi.2021.648531

**Published:** 2021-04-20

**Authors:** Wen-biao Xian, Xin-chong Shi, Gan-hua Luo, Chang Yi, Xiang-song Zhang, Zhong Pei

**Affiliations:** ^1^Department of Neurology, The First Affiliated Hospital, Sun Yat-sen University, Guangzhou, China; ^2^Guangdong Provincial Key Laboratory of Diagnosis and Treatment of Major Neurological Diseases, National Key Clinical Department and Key Discipline of Neurology, Guangzhou, China; ^3^Department of Nuclear Medicine, The First Affiliated Hospital, Sun Yat-sen University, Guangzhou, China

**Keywords:** Parkinson's disease, mutiple system atrophy, PET, FDG, F-DOPA

## Abstract

It is difficult to differentiate between Parkinson's disease and multiple system atrophy parkinsonian subtype (MSA-P) because of the overlap of their signs and symptoms. Enormous efforts have been made to develop positron emission tomography (PET) imaging to differentiate these diseases. This study aimed to investigate the co-registration analysis of ^18^F-fluorodopa and ^18^F-flurodeoxyglucose PET images to visualize the difference between Parkinson's disease and MSA-P. We enrolled 29 Parkinson's disease patients, 28 MSA-P patients, and 10 healthy controls, who underwent both ^18^F-fluorodopa and ^18^F-flurodeoxyglucose PET scans. Patients with Parkinson's disease and MSA-P exhibited reduced bilateral striatal ^18^F-fluorodopa uptake (*p* < 0.05, vs. healthy controls). Both regional specific uptake ratio analysis and statistical parametric mapping analysis of ^18^F-flurodeoxyglucose PET revealed hypometabolism in the bilateral putamen of MSA-P patients and hypermetabolism in the bilateral putamen of Parkinson's disease patients. There was a significant positive correlation between ^18^F-flurodeoxyglucose uptake and ^18^F-fluorodopa uptake in the contralateral posterior putamen of MSA-P patients (*r*s = 0.558, *p* = 0.002). Both ^18^F-flurodeoxyglucose and ^18^F-fluorodopa PET images showed that the striatum was rabbit-shaped in the healthy control group segmentation analysis. A defective rabbit-shaped striatum was observed in the ^18^F-fluorodopa PET image of patients with Parkinson's disease and MSA-P. In the segmentation analysis of ^18^F-flurodeoxyglucose PET image, an intact rabbit-shaped striatum was observed in Parkinson's disease patients, whereas a defective rabbit-shaped striatum was observed in MSA-P patients. These findings suggest that there were significant differences in the co-registration analysis of ^18^F-flurodeoxyglucose and ^18^F-fluorodopa PET images, which could be used in the individual analysis to differentiate Parkinson's disease from MSA-P.

## Introduction

The diagnosis of Parkinson's disease (PD) is mainly based on the typical clinical presentation of cardinal signs and its being well-responsive to levodopa treatment (Armstrong and Okun, 2020); however, misdiagnosis of PD is not uncommon. The most common misdiagnoses are related to parkinsonism-plus syndromes, such as multiple system atrophy (MSA), which could be classified as the parkinsonian subtype (MSA-P) with predominant parkinsonism, and the cerebellar subtype (MSA-C) with predominant cerebellar features (Koga and Dickson, 2018). Asymmetry of the cardinal features of parkinsonism and good response to levodopa, which are characteristic manifestations associated with PD, could also be seen in some patients with MSA-P (Jost et al., 2019), which makes it difficult to make the differential diagnosis on the basis of clinical criteria alone. In clinical pathological studies, only 76% of postmortem-confirmed cases were diagnosed correctly as PD (Rajput et al., 1991), while 55% of postmortem-confirmed MSA cases were misdiagnosed (Litvan et al., 1997).

Many objective methods have been tested for the differential diagnosis between PD and MSA-P, such as serum neuronal exosomes (Jiang et al., 2020), proteins in cerebrospinal fluid (Singer et al., 2020), and neuroimaging (Saeed et al., 2020). Multiple imaging modalities such as magnetic resonance imaging (MRI) and positron emission tomography (PET) have been increasingly used to investigate the morphologic and functional characteristics of MSA, PD, and other atypical parkinsonisms (Zhao et al., 2020). The second consensus statement on the diagnosis of MSA (Gilman et al., 2008) and the recent Movement Disorders Society clinical diagnostic criteria for PD (Postuma et al., 2015) have included the results of a few of these neuroimaging techniques to serve as supportive criteria or exclusion criteria for the diagnosis of diseases. Thus, cerebral MRI and PET may aid in an early, accurate, and objective diagnostic classification by highlighting the underlying neurochemical and neuroanatomical changes that underlie the spectrum of disorders.

The differential diagnosis can be assisted by the characteristic features of MSA revealed by MRI, such as putaminal hyperintensive rim and “hot cross bun” sign of the pons (Hughes et al., 2002; Poewe and Wenning, 2002). However, putaminal hyperintensive rim occurs in only 31.8% of MSA-P patients and is also present in 6.7% of PD patients (Zhao et al., 2020). The “hot cross bun” sign exhibits the highest specificity in MSA-C patients but is not found in MSA-P patients (Zhao et al., 2020). In PET studies, ^18^F-fluorodopa (^18^F-Dopa) can only differentiate healthy subjects from parkinsonism patients, but it is inadequate for differentiating between MSA-P and PD because nigrostriatal dopaminergic neurons degenerate in these disorders (Burn et al., 1994). Imaging disease-specific patterns of regional glucose metabolism with ^18^F-flurodeoxyglucose (^18^F-FDG) PET allow for a highly accurate distinction between PD and MSA, such as PD-related pattern (Matthews et al., 2018) and MSA-related pattern (Akdemir et al., 2014). Due to the low spatial resolution of FDG PET images, visual analysis is difficult to detect significant anomalies; hence, these disease-specific patterns are calculated by voxel-based statistical analysis in comparison with healthy control subjects with statistical parametric mapping (SPM) software (Ko et al., 2017). SPM is useful in the group analysis of FDG PET images but is hampered in individual diagnosis. Single-patient SPM analysis could be also used, but this statistical method is controversial because only one patient is included in one group of the data. Single SPM analysis only derives a calculated image, which may differ from the actual brain structure of the patient; thus, the result could be affected by the selection of a normal control group. Variations in PET scanners and image reconstruction algorithms have been shown to systematically shift image quality in SPM analysis (Kogan et al., 2019).

We hypothesize that there are disease-specific patterns related to PD and MSA-P in both ^18^F-Dopa and ^18^F-FDG PET, and the two kinds of imaging modalities have their own advantages and disadvantages. In the present study, we used the co-registration analysis of ^18^F-Dopa and ^18^F-FDG PET to visualize the characteristics of the images without any calculation or image distortion, which could be easily used in the individual differential diagnosis between PD and MSA-P.

## Materials and Methods

### Subjects and Clinical Assessments

Twenty-nine consecutive PD patients (mean age 57.0 ± 10.0 years), 28 MSA-P patients (mean age 60.6 ± 5.9 years), and 10 healthy controls (HCs, mean age 59.0 ± 8.2 years) were enrolled in this study. The PD patients were diagnosed according to Movement Disorders Society clinical diagnostic criteria for PD (Postuma et al., 2015), and MSA patients were diagnosed according to the second consensus statement on the diagnosis of MSA (Gilman et al., 2008). The patients were at a relatively early stage of disease: disease duration of MSA-P patients was within 5 years; Hoehn and Yahr stage (H&Y) of PD patients ranged from stage 1 to stage 3. Age-matched HCs without neurological disorders were also enrolled. The exclusion criteria for all participants were as follows: Patients with drug-induced parkinsonism, vascular parkinsonism, progressive supranuclear paralysis, corticobasal degeneration, and dementia with Lewy bodies; prior brain surgery, including deep brain stimulation; and other neurologic diseases, including head trauma, stroke, and brain tumor. Written informed consent was obtained from all participants in accordance with the Declaration of Helsinki, and the study was approved by the local Ethical Committee of the First Affiliated Hospital of Sun Yat-sen University.

Disease severity of the patients was measured with H&Y stage. Functional independence was measured using the Schwab and England Activities of Daily Living Scale (SEADL). Mini-Mental State Examination score and disease duration were also recorded. Patients in the PD group consisted of 16 males and 13 females, with a disease duration of 4.1 ± 1.7 years, H&Y stage 2.0 ± 0.6, Mini-Mental State Examination score of 28.6 ± 6.2, and SEADL score of 79.7 ± 6.7. Patients in the MSA-P group consisted of 16 males and 12 females, with a disease duration of 3.3 ± 1.0 years, H&Y stage 3.3 ± 0.7, Mini-Mental State Examination score of 26.6 ± 1.1, and SEADL score of 72.0 ± 3.8. There was no significant difference between the PD and the MSA-P groups, except a more severe H&Y stage and a reduced SEADL score in the MSA-P group (*p* < 0.05).

### PET Imaging

All participants underwent both cerebral ^18^F-FDG and ^18^F-Dopa PET examination using a Gemini GXL 16 PET/CT scanner (Philips, Amsterdam, the Netherlands) using the three-dimensional (3D) acquisition mode, with dopamine receptor agonists withdrawn for at least 72 h and levodopa withdrawn for at least 12 h. Participants were fasted for at least 8 h before ^18^F-FDG PET imaging. Subjects lay comfortably in a supine position in a room with dimmed lighting and low background noise, to minimize any sympathetic response. A low-dose computerized tomography (CT, 120 KV, 250 mA) about 45 min after intravenous injection of ^18^F-FDG (185 MBq) PET was performed immediately after CT for about 10 min. Transversal PET slices were reconstructed by means of CT-based attenuation correction using an iterative algorithm. ^18^F-Dopa PET was performed on another day and the subjects fasted for at least 4 h. They were given oral entacapone (200 mg) 1 h prior to the injection of 370–444 MBq of ^18^F-Dopa (Ruottinen et al., 1995). Each participant had a quiet rest lasting 90 min before a 10-min PET emission acquisition. PET images were reconstructed with low-dose CT images for attenuation correction.

### Image Analysis

All the PET image data were processed and analyzed using the software MIPAV (version 7.0.1, US Department of Health and Human Services). The striatal regions of interest (ROIs) of PET images were defined using a technique developed at our institution (Shi et al., 2019). To accurately analyze the imaging profile characteristics of the subjects, we divided the striatum into anterior/posterior putamen (APu/PPu) and caudate nucleus in both ^18^F-FDG and ^18^F-Dopa PET image data. Specific uptake ratio (SUR) was calculated to evaluate the radioisotopic activity in the selected ROIs. The occipital cortex was regarded as the reference region in the ^18^F-Dopa PET image and the average value of the whole brain was regarded as the reference region in ^18^F-FDG PET image for calculating SUR by (target uptake—reference uptake)/reference uptake. In patients with PD and MSA-P, the contralateral ROIs were considered to be the opposite brain regions in relation to the predominantly affected limbs. The SUR was calculated by two expert radiologists (XS and XZ) who were blinded to participants' clinical data and co-registration analysis.

### Image Co-registration Processing

**Figure 1:**
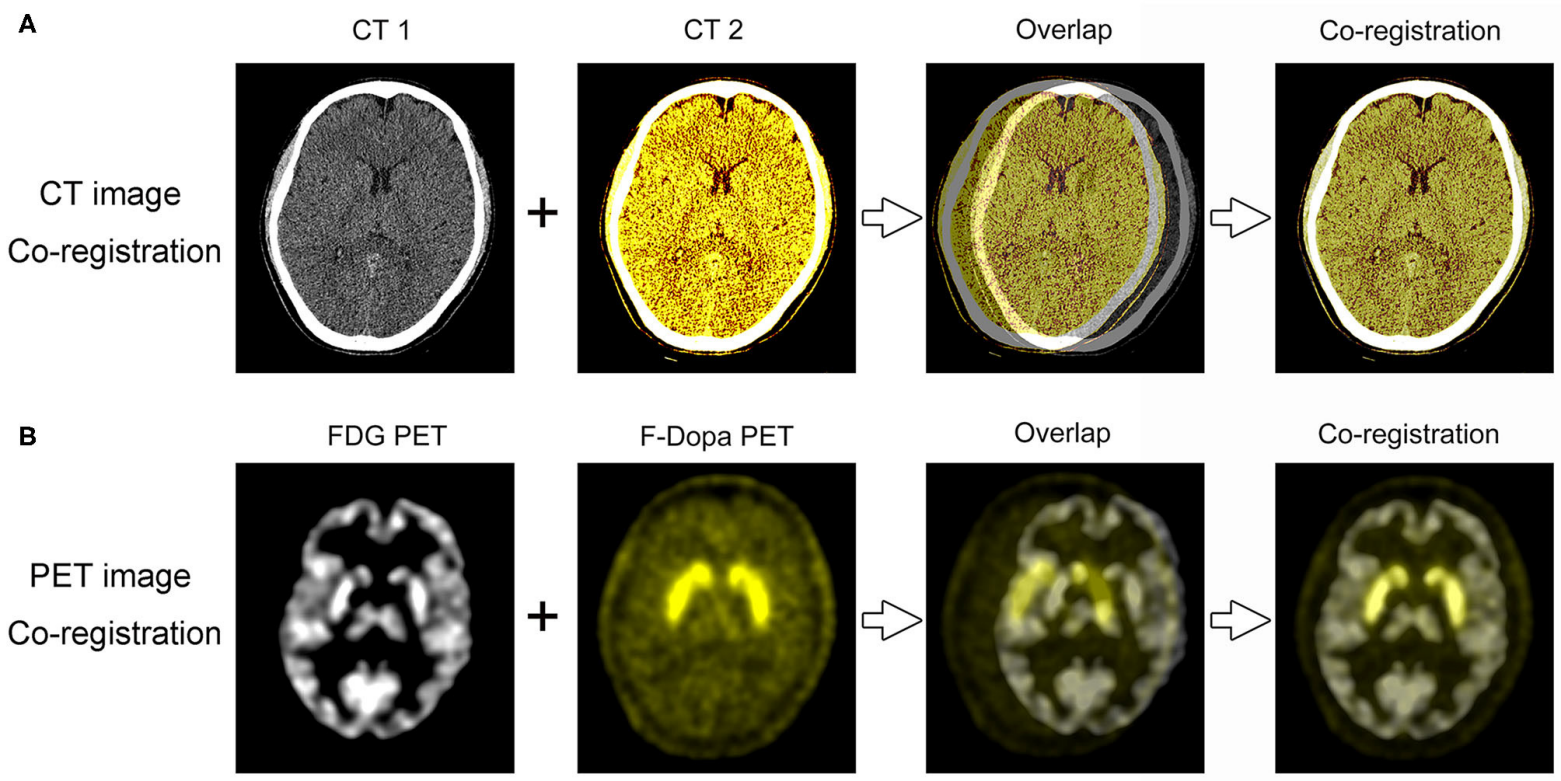
PET/CT image co-registration processing. **(A)** The first step was to make the co-registration of two CT images using 3D Slicer software. **(B)** The second step was to make automatic registration of the ^18^F-FDG and ^18^F-Dopa PET images.

The co-registration of three-dimensional PET and CT images was analyzed using software 3D Slicer (version 4.10.0, www.slicer.org). The 3D Slicer is a multiplatform developed to perform an interactive semiautomatic segmentation and image registration that allows the quantitation of features of interest from medical imaging including MRI, CT, and nuclear medicine. The first step was to make the co-registration of two CT images with a higher image resolution than PET, using a landmark registration algorithm of the software (Figure 1A). The second step was to use the “transforms” tool to make automatic registration of the ^18^F-FDG and ^18^F-Dopa PET images (Figure 1B). Segmentation of striatal ROIs was accomplished by two methods: (1) Automatic brain structure segmentation. (2) Manual segmentation: an incorporated voxel made by automatic segmentation required a manual revision of the pixels, slice by slice, selected for a more precise segmentation. Segmentation of striatal ROIs in ^18^F-FDG and ^18^F-Dopa PET images was labeled with different colors to present the images more hierarchically. Finally, a 3D model was developed using the Model Maker to visualize the morphological structure of striatal ROIs. When the two models from ^18^F-FDG and ^18^F-Dopa PET overlapped in the co-registration analysis, the opacity of one model was reduced to 50% to visualize the 3D image more clearly. The co-registration processing was performed by two investigators (GL and CY) who were blinded to participants' clinical data and SUR analysis.

### SPM Analysis

SPM analysis was performed using SPM5 (Wellcome Department of Cognitive Neurology, Institute of Neurology, University College London). To detect any regional metabolic pattern by SPM, all the images were analyzed using the appropriate voxel-by-voxel unvaried statistical tests of group comparisons (two-sample *t-*test, *p* < 0.05) within the two groups (PD vs. HC, MSA-P vs. HC). The results were displayed on the three orthogonal planes of an MRI.

### Statistical Analysis

All analyses were conducted using SPSS 19.0 software (Version 19.0., IBM Corp., Armonk, NY, United States). Continuous data were given as mean ± standard deviation (SD); categorical data were given as frequencies or percentages. A Kruskal–Wallis test was applied to compare the mean differences between PD, MSA-P, and HC groups. The significant correlations between the SUR values in striatal ROIs of ^18^F-FDG and ^18^F-Dopa were evaluated using a Spearman's test. The statistical significance was set at *p* < 0.05.

## Results

### Regional PET Analyses

In comparison with the HC group, significant reduction of ^18^F-Dopa uptake was observed in the bilateral putamen and caudate nucleus in patients in both PD and MSA-P groups (Table 1). The reduction of ^18^F-Dopa uptake in the putamen was more serious in the posterior than the anterior area, but there was no significant difference in the PPu/APu ratio of SUR values between PD and MSA-P groups. Significant differences in ^18^F-FDG uptake were observed between PD, MSA-P, and HC groups. In comparison with the HC group, there was a significant increase in ^18^F-Dopa uptake in the bilateral caudate nucleus and putamen of patients in the PD group. On the contrary, the ^18^F-FDG uptake was significantly reduced in the bilateral putamen of patients in the MSA-P group when compared with the HC group. *Post-hoc* Bonferroni tests showed that the reduction of ^18^F-FDG uptake in the putamen was more serious in the posterior than the anterior area in MSA-P patients compared to the PD and HC groups. The Spearman correlation analysis demonstrated a significant positive correlation of ^18^F-FDG uptake with ^18^F-Dopa uptake in the contralateral posterior putamen in MSA-P patients (*r*s = 0.558, *p* = 0.002). There was no significant correlation of tracer uptake between ^18^F-FDG and ^18^F-Dopa in other parts of the brain.

**Table 1:** Specific uptake ratios of PET image of all participants.

**Region**	**PD**	**MSA-P**	**HC**	***p***
^**18**^**F-Dopa PET**				
Contralateral Ca	2.25 ± 0.54^c^	2.31 ± 0.36^c^	3.00 ± 0.41^ab^	<0.001
Ipsilateral Ca	2.34 ± 0.61^c^	2.35 ± 0.40^c^	3.00 ± 0.45^ab^	0.003
Contralateral APu	2.27 ± 0.58^c^	2.26 ± 0.36^c^	3.83 ± 0.51^ab^	<0.001
Ipsilateral APu	2.37 ± 0.58^c^	2.37 ± 0.47^c^	3.67 ± 0.58^ab^	<0.001
Contralateral PPu	1.80 ± 0.32^c^	1.74 ± 0.31^c^	3.77 ± 0.62^ab^	<0.001
Ipsilateral PPu	1.88 ± 0.36^c^	1.92 ± 0.40^c^	3.68 ± 0.61^ab^	<0.001
Contralateral PPu/APu	0.81 ± 0.12^c^	0.77 ± 0.10^c^	0.96 ± 0.05^ab^	<0.001
Ipsilateral PPu/APu	0.81 ± 0.09^c^	0.79 ± 0.11^c^	0.98 ± 0.10^ab^	<0.001
^**18**^**F-FDG PET**				
Contralateral Ca	2.12 ± 0.18^c^	2.09 ± 0.22^c^	1.89 ± 0.15^ab^	0.007
Ipsilateral Ca	2.10 ± 0.20^c^	2.15 ± 0.18^c^	1.84 ± 0.18^ab^	0.001
Contralateral APu	2.74 ± 0.30^bc^	2.24 ± 0.22^a^	2.36 ± 0.22^a^	<0.001
Ipsilateral APu	2.72 ± 0.31^bc^	2.42 ± 0.21^a^	2.37 ± 0.15^a^	<0.001
Contralateral PPu	2.86 ± 0.32^bc^	1.71 ± 0.24^ac^	2.28 ± 0.13^ab^	<0.001
Ipsilateral PPu	2.91 ± 0.36^bc^	2.00 ± 0.32^ac^	2.37 ± 0.20^ab^	<0.001
Contralateral PPu/APu	1.05 ± 0.06^b^	0.76 ± 0.07^ac^	0.97 ± 0.05^b^	<0.001
Ipsilateral PPu/APu	1.07 ± 0.06^b^	0.83 ± 0.12^ac^	1.00 ± 0.05^b^	<0.001

*PD, Parkinson's disease; MSA-P, multiple system atrophy parkinsonian subtype; HC, healthy control; FDG, flurodeoxyglucose; Pu, putamen; Ca, caudate nucleus; Apu, anterior putamen; PPu, posterior putamen*.

*p < 0.01 compared to ^a^PD, ^b^MSA-P, and ^c^HC*.

### PET Image Segmentation Analysis

**Figure 2:**
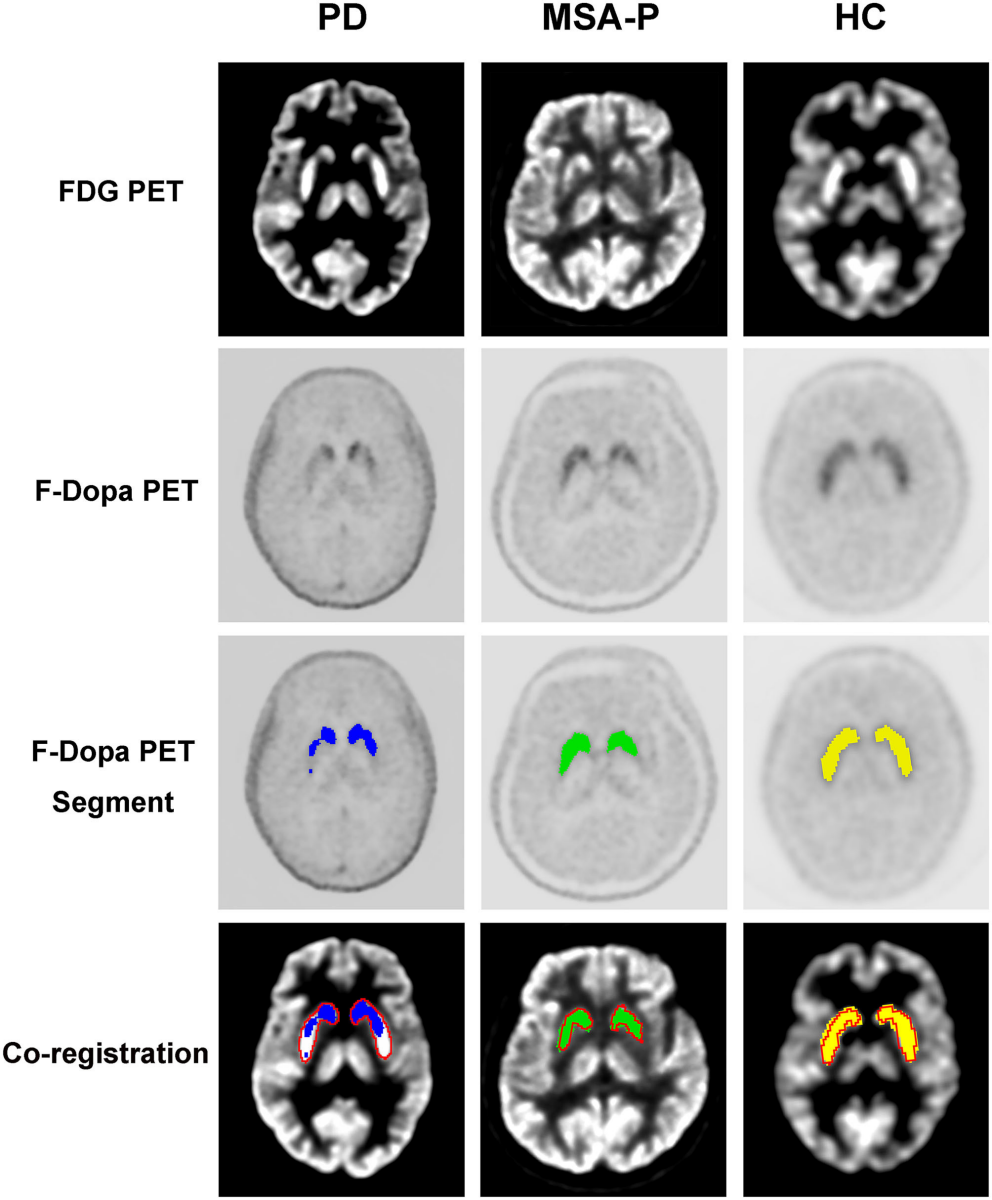
Segmentation of striatal PET images of participants. The striatal segments of ^18^F-Dopa PET (PD: blue color; MSA-P: green color; HC: yellow color) and ^18^F-FDG PET (red color) images were labeled with different colors. Co-registration analysis showed that the segments of striatum in ^18^F-FDG and ^18^F-Dopa PET of PD patients were mismatched, with intact striatal segments in ^18^F-FDG PET and damaged striatal segments in^18^F-Dopa PET. The segments of striatum in MSA-P and HC groups were matched in ^18^F-FDG and ^18^F-Dopa PET images. The striatal segments were defective in MSA-P patients, whereas intact in the HC group.

**Figure 3:**
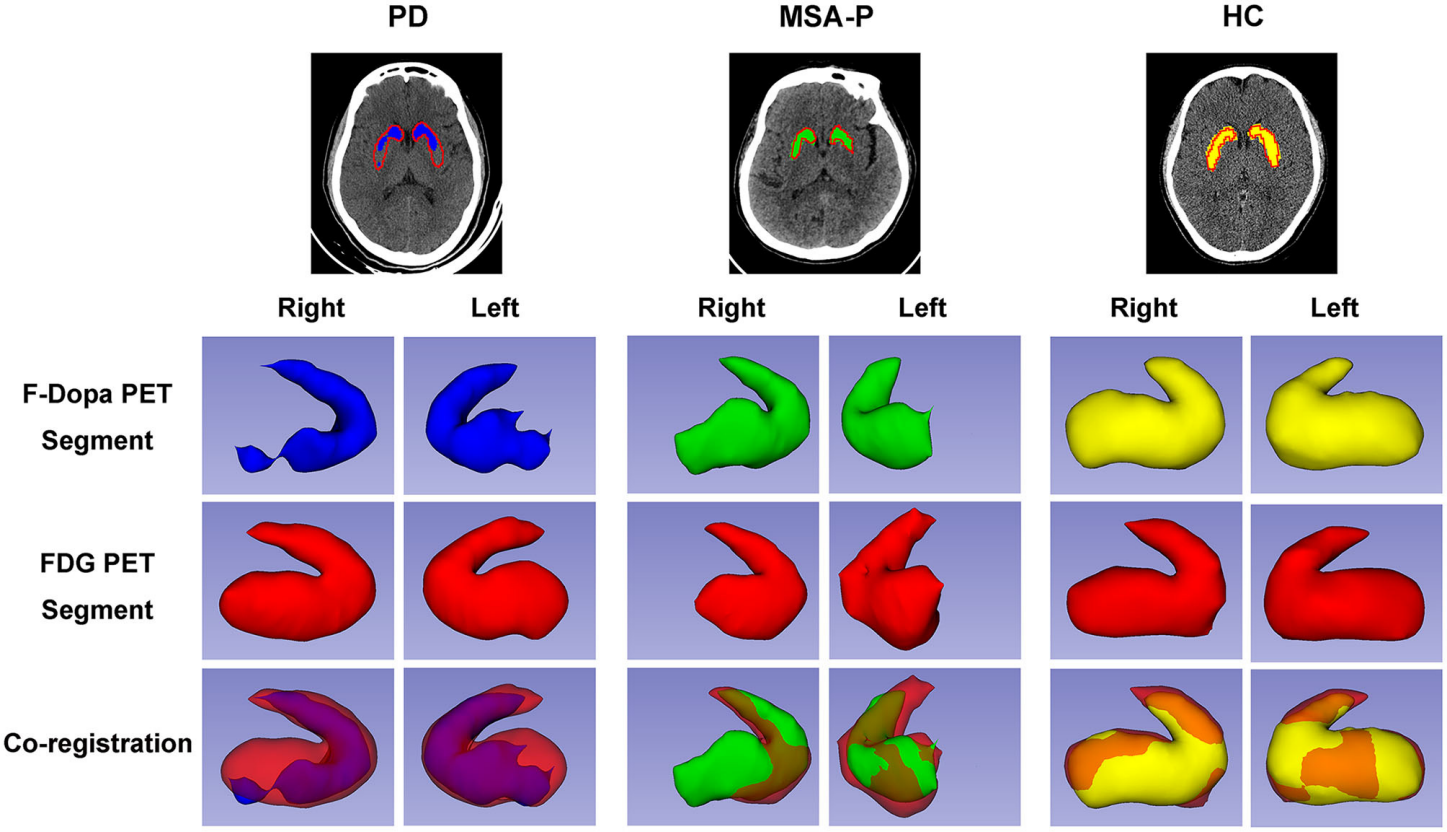
Three-dimensional visualization of striatal PET images of participants. The striatal segments of ^18^F-Dopa PET (PD: blue color; MSA-P: green color; HC: yellow color) and ^18^F-FDG PET (red color) images were labeled with different colors. Both ^18^F-FDG and ^18^F-Dopa PET images showed that the striatum had a rabbit-shaped appearance in the HC group. A defective rabbit-shaped striatum was shown in the ^18^F-Dopa PET image of patients with PD and MSA-P. In the ^18^F-FDG PET image, an intact rabbit-shaped striatum was shown in PD patients, whereas a defective rabbit-shaped striatum was shown in MSA-P patients.

The graphic comparison of the segmentations of striatal PET images between PD, MSA-P, and HC groups is shown in Figures 2, 3. In the HC group, both ^18^F-FDG and ^18^F-Dopa PET images showed that the striatum had a full rabbit-shaped appearance in the segmentation analysis. In ^18^F-Dopa PET image, the normal appearance of the striatum of patients in the PD and MSA-P groups was not completely displayed, and there were mainly graphic defects in the posterior part of the putamen. In ^18^F-FDG PET image, the rabbit-shaped appearance of the striatum segment in PD patients was not impaired, whereas in MSA-P patients, there were significant defects of bilateral striatum segments in the posterior putamen. Co-registration image analysis showed that the segments of the striatum in ^18^F-FDG and ^18^F-Dopa PET of PD patients were mismatched, with intact striatal segments in ^18^F-FDG PET but damaged striatal segments in ^18^F-Dopa PET. The segments of the striatum in MSA-P and HC groups were matched in ^18^F-FDG and ^18^F-Dopa PET images. The striatal segments were defective in MSA-P patients but intact in the HC group. A significant difference was observed in the frequency of rabbit-shaped striatal segments in the segmentation analysis between PD, MSA-P, and HC groups (Table 2).

**Table 2:** PET image segmentation analysis of all participants.

**Striatal segment**	**PD (*n* = 29)**		**MSA-P (*n* = 28)**		**HC (*n* = 10)**	
	**^18^F-FDG**	**^18^F-Dopa**	**^18^F-FDG**	**^18^F-Dopa**	**^18^F-FDG**	**^18^F-Dopa**
Intact rabbit-shaped	29	0	0	0	10	10
Defective rabbit-shaped	0	29	28	28	0	0

*PD, Parkinson's disease; MSA-P, multiple system atrophy parkinsonian subtype; HC, healthy control; FDG, flurodeoxyglucose*.

### SPM Analysis of ^18^F-FDG PET

**Figure 4:**
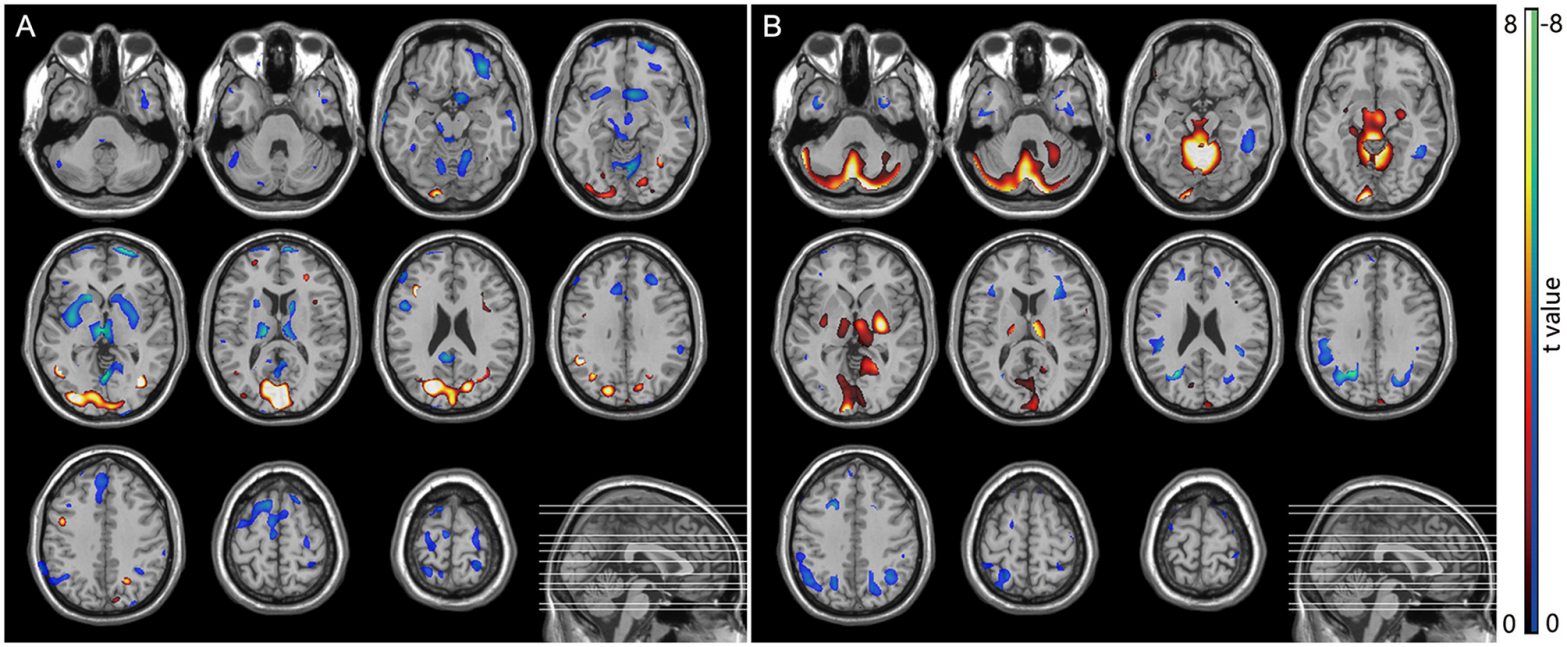
SPM analysis of 18F-FDG PET. **(A)** MSA patients vs. normal controls showed hypometabolism in the bilateral putamen. **(B)** PD patients vs. normal controls showed hypermetabolism in the bilateral putamen, cerebellum, and occipital lobes, along with hypometabolism in the frontal, parietal, postcentral cortices, and cingulate areas.

In the MSA-P group, a significant glucose hypometabolism in the bilateral putamen was detected as compared with the HC group by statistical voxel-based analysis (Figure 4A). In comparison with the HC group, the PD patients were found to have a significant glucose hypermetabolism in the bilateral putamen, cerebellum, and occipital lobes, along with hypometabolism in the frontal, parietal, postcentral cortices, and cingulate areas (Figure 4B).

## Discussion

This study is the first to demonstrate the diagnostic value of co-registration analysis of ^18^F-FDG and ^18^F-Dopa PET for differentiating MSA-P from PD. Segmentation analysis of the rabbit-shaped appearance of the striatum segment of PET images not only could distinguish PD and MSA-P patients from healthy controls but also further differentiated PD from MSA-P. The segmentation analysis has also been verified by the traditional regional PET uptake value analysis and the latest SPM analysis used for identifying disease-specific metabolic patterns.

The segmentation analysis showed a full rabbit-shaped appearance of the striatal segment of PET images in both ^18^F-Dopa and ^18^F-FDG PET in the HC group, indicating intact integrity of the brain dopamine system and preserved function of the basal ganglia-related pathway. In patients with PD, a defective striatal segment was shown in ^18^F-Dopa PET due to the degeneration of nigrostriatal dopaminergic neurons. However, the rabbit-shaped appearance of the striatal segment was intact in the ^18^F-Dopa PET image, indicating that the postsynaptic function of the dopaminergic neurons and related pathways were not damaged (Ko et al., 2018; Shine et al., 2019). Defective striatal segments were shown in both ^18^F-Dopa and ^18^F-FDG PET images in patients with MSA-P, which is consistent with neuropathologic features of MSA (Yoshida, 2007; Jellinger, 2014). A neuropathologic study on MSA showed that the glial cytoplasmic inclusions, neuronal nuclear inclusions, and diffuse homogeneous alpha-synuclein staining in neuronal nuclei and cytoplasm were distributed widely in lesions in the pontine nuclei, putamen, substantia nigra, and other parts of the brain, indicating that both presynaptic function (detected by ^18^F-Dopa PET) and postsynaptic pathway (detected by ^18^F-FDG PET) of dopaminergic neurons were impaired.

The segmentation analysis could be verified by regional PET uptake value analysis. A significant reduction of ^18^F-Dopa uptake was observed in the bilateral striatum of patients with PD and MSA-P. There was no significant difference in ^18^F-Dopa uptake in the bilateral striatum between patients with PD and MSA-P, which is consistent with previous studies (Burn et al., 1994; Hu et al., 2002). The ^18^F-Dopa and other presynaptic dopaminergic radiotracers of PET or single-photon emission computed tomography are useful to separate healthy subjects from Parkinsonism patients, but they are inadequate for differentiating between MSA-P and PD (Perju-Dumbrava et al., 2012; Kaasinen et al., 2019). The further ^18^F-FDG PET showed a significant decrease in the bilateral putamen of patients with MSA-P and a significant increase in ^18^F-FDG uptake in the bilateral striatum of patients with PD. The diametrically opposite results suggest that the pathogeneses of the two diseases are different. ^18^F-FDG PET measures cerebral glucose metabolism, which reflects synaptic and neuronal activity. Loss of striatal dopamine in PD leads to changes of activity in both the direct and indirect striatal pathways, which could be detected by the ^18^F-FDG PET. The primary pathological abnormality in PD is confined to the substantia nigra, and the degeneration of dopaminergic projection neurons from the substantia nigra to the striatum results in widespread alterations in the functional activity (Alexander et al., 1990; Parent and Hazrati, 1995) of the basal ganglia. The loss of inhibitory dopaminergic input to the striatum results in increased activity of the striatum, which is shown in striatal hypermetabolism in ^18^F-FDG PET in patients with PD. The metabolic patterns in MSA-P represent regional postsynaptic neuronal loss directly related to abnormal protein deposition that characterizes the neurodegenerative disease (Tripathi et al., 2013). A susceptibility-weighted imaging study showed iron deposition in the putamen of patients with MSA-P caused by iron-mediated oxidative stress, which might appear as glucose hypometabolism in ^18^F-FDG PET, which could differentiate MSA-P from PD (Yoon et al., 2015). We also observed a significant positive correlation of ^18^F-FDG uptake with ^18^F-Dopa uptake in the contralateral posterior putamen in MSA-P patients in our study, indicating that presynaptic function and postsynaptic pathway of dopaminergic neurons are synchronously damaged in MSA-P. In addition, regarding the similar disease duration, MSA-P patients in our study exhibited higher severity than PD patients with higher H&Y score, which is consistent with previous PET studies (Schönecker et al., 2019; Zhao et al., 2020), supporting the fact that MSA-P develops more rapidly than PD. PET studies showed that striatal uptake of ^18^F-Dopa significantly correlated with the H&Y stage in both patients with PD (Eshuis et al., 2006) and MSA (Taniwaki et al., 2002). However, ^18^F-FDG PET exhibited an opposite pattern of 18F-FDG uptake in the striatum between PD and MSA. For example, a longitudinal FDG-PET study showed that, as evidenced by PD-related pattern activity, a metabolic network characterized by hypermetabolism in the putamen increased linearly with disease progression of PD (Huang et al., 2007). In contrast, MSA-related pattern, a metabolic network characterized by hypometabolism in the putamen, has been shown to have a positive correlation with disease duration in patients with MSA (Ko et al., 2017). Collectively, these studies suggest that with the progression of the disease, the striatal metabolism in PD patients increases, whereas the striatal metabolism in MSA patients decreases. Therefore, H&Y stage scores do not affect the differential diagnosis of the two diseases in our co-registration analysis of PET images.

Our SPM analysis also confirmed the results of the above segmentation analysis and ^18^F-FDG uptake changes in patients with PD and MSA-P. PD patients were found to have a significant glucose hypermetabolism in the bilateral putamen, cerebellum, and occipital lobes, and a significant glucose hypometabolism in the bilateral putamen was detected in patients with MSA-P. SPM analysis can clearly differentiate between these two forms of parkinsonism: the glucose metabolism in the putamen is preserved or elevated in PD but significantly decreased in MSA-P, which is useful in the group analysis of FDG PET image. Our results are consistent with previous studies (Ma et al., 2007; Meles et al., 2020). However, the widespread implementation of such cerebral disease-related metabolic patterns in multicenter collaborations and clinical practice has been hindered by differences between PET scanners as well as reconstruction algorithms (Kogan et al., 2019). For example, hypermetabolism in the putamen was shown in the abnormal metabolic network in PD (Ma et al., 2007; Meles et al., 2020), but there was no significant change in metabolism of the putamen in the PD-related pattern analysis in several FDG PET studies (Holtbernd et al., 2015; Ko et al., 2017). The selection of a normal control group with differing ages, genders, and ethnicities could also cause potential bias in the SPM analysis (Kogan et al., 2019). It is interesting that significant glucose hypometabolism in the bilateral putamen can be found in MSA-P patients compared with healthy controls, but SPM analysis did not show reduced cerebellar metabolism. Our result is consistent with previous studies (Zhao et al., 2012, 2020), and the character of FDG PET image is consistent with the neuropathologic feature of MSA-P (predominated in basal ganglia) and MSA-C (predominated in cerebellum) (Ozawa et al., 2004).

There were several limitations in this study. First, our study sample was relatively small and longitudinal; hence, studies with a large sample size are needed in the future. Second, single-patient SPM analysis was not performed because there would have been the need to change the age-matched HC group from a large database if the patient's age changed. Finally, other types of parkinsonism-plus syndromes, such as progressive supranuclear paralysis and corticobasal degeneration, were not included in the study.

In conclusion, the co-registration analysis of ^18^F-FDG and ^18^F-Dopa PET demonstrated great diagnostic values for differentiating MSA-P from PD, which could be easily used in the individual analysis in clinics. Further studies are needed to evaluate the segmentation analysis in the differential diagnosis of other atypical Parkinsonisms, such as progressive supranuclear paralysis and corticobasal degeneration.

## Data Availability Statement

The raw data supporting the conclusions of this article will be made available by the authors, without undue reservation.

## Ethics Statement

The studies involving human participants were reviewed and approved by local Ethical Committee of the First Affiliated Hospital of Sun Yat-sen University. The patients/participants provided their written informed consent to participate in this study.

## Author Contributions

W-bX: conceptualization, data curation and analysis, validation, and writing original draft. X-cS: data curation and analysis, validation, and writing original draft. G-hL and CY: data curation and analysis, and validation. X-sZ and ZP: project administration, resources, supervision, data analysis, review, and editing. All the authors read and approved the final manuscript.

## Conflict of Interest

The authors declare that the research was conducted in the absence of any commercial or financial relationships that could be construed as a potential conflict of interest.
